# Association of Circulating Cathepsin B Levels With Blood Pressure and Aortic Dilation

**DOI:** 10.3389/fcvm.2022.762468

**Published:** 2022-03-29

**Authors:** Tianci Chai, Mengyue Tian, Xiaojie Yang, Zhihuang Qiu, Xinjian Lin, Liangwan Chen

**Affiliations:** ^1^Department of Cardiovasclar Surgery, Fujian Medical University Union Hospital, Fuzhou, China; ^2^Key Laboratory of Cardio-Thoracic Surgery (Fujian Medical University), Fujian Province University, Fuzhou, China; ^3^Department of Anesthesiology, Xinyi People’s Hospital, Xuzhou, China; ^4^Key Laboratory of Ministry of Education for Gastrointestinal Cancer, The School of Basic Medical Sciences, Fujian Medical University, Fuzhou, China; ^5^Department of Thoracic Surgery, Fujian Medical University Union Hospital, Fuzhou, China

**Keywords:** blood pressure, genome-wide association study, circulating proteins, cathepsin, spontaneous coronary artery dissection (SCAD), aortic dissection (AD)

## Abstract

Hypertension is a key risk factor for spontaneous coronary artery dissection (SCAD) and aortic dilation. Circulating proteins play key roles in a range of biological processes and represent a major source of druggable targets. The aim of this study was to identify circulating proteins that were associated with blood pressure (BP), SCAD and aortic dilation. We identified shared genetic variants of BP and SCAD in genome-wide association studies, searched for circulating protein affected by these variants and examined the association of circulating protein levels with BP, aortic aneurysm and dissection (AAD) and aortic diameters by integrating data from circulating protein quantitative trait loci (pQTL) studies and genome wide association study (GWAS) in individuals from the UK Biobank using two-sample Mendelian randomization analysis methods. Single nucleotide polymorphisms (SNPs) in *JAG1*, *ERI1*, *ULK4*, *THSD4*, *CMIP*, *COL4A2*, *FBN1*, *FAM76B*, *FGGY*, *NUS1*, and *HNF4G*, which were related to extracellular matrix components, were associated with both BP and SCAD. We found 49 significant pQTL signals among these SNPs. The regulated proteins were encoded by *MMP10*, *IL6R*, *FIGF*, *MMP1*, *CTSB*, *IGHG1*, *DSG2*, *TTC17*, *RETN*, *POMC*, *SCARF2*, *RELT*, and *GALNT16*, which were enriched in biological processes such as collagen metabolic process and multicellular organism metabolic process. Causal associations between BP and AAD and aortic diameters were detected. Significant associations between circulating levels of cathepsin B, a well-known prorenin processing enzyme, and BP and aortic diameters were identified by using several Mendelian randomization analysis methods and were validated by independent data.

**Conclusion:**

The present study identified the association between circulating cathepsin B and BP and aortic diameters. The findings indicated that BP-associated genetic variants may influence aortic dilation risk by circulating proteins that regulate BP.

## Introduction

Aortic dissection (AD) is the major diseases affecting the aorta and a leading cause of morbidity and mortality. Recently, the Global Burden Disease 2010 project demonstrated that the overall global death rate from aortic aneurysms and AD increased from 2.49 per 100,000 to 2.78 per 100,000 inhabitants between 1990 and 2010 (1, 2). The burden increases with age, and men are more often affected than women (1). Spontaneous coronary artery dissection (SCAD) is one of the most dangerous forms of vascular disease and is characterized by endometrial rupture and intramural hematoma formation. Until now, the occurrence and development of SCAD and AD have been unpredictable.

Aortic disease in Marfan syndrome is the result of defects in the fibrillin-1 (*FBN1*) gene that localizes to chromosome 15q15-31 (3). Genetic studies have demonstrated familial aggregation of AD and have resulted in the mapping of several novel genetic loci for the condition (4–6). Genes associated with AD include *FBN1*, *TGFBR1/2*, *TGFB1*, *EFEMP2*, *MYH11*, *ACTA2*, *COL3A1*, *SLC2A10*, *SMAD3*, *LOX*, *FOXE3*, *MFAP5*, *MAT2A*, *PRKG1*, and so on (7). Genetic variants in *LRP1* and *ULK4* are associated with sporadic thoracic AD (5).

The common occurrence of SCAD in young patients without risk factors for atherosclerosis and the identification of cases with familial association have led to the hypothesis of possible genetically mediated pathophysiologic mechanisms. Pathogenic variants were found in *COL3A1* (8), *SMAD3* (8, 9), *TGFBR1/2*, *TGFB 2/3* (10), *FBN1*, *PHACTR1/EDN1* (11), *TSR1* (12, 13), *TLN1* (14), and others (14–16). A recent genome wide association study (GWAS) has identified associations at chromosome 1q21.2 in *ADAMTSL4*, chromosome 6p24.1 in *PHACTR1* and chromosome 12q13.3 in *LRP1* and identified associations of non-coding variants in the *LRP1* gene at chromosome 12q13.3 and near *MRP6/KCNE3* at chromosome 21q22.11 for SCAD (15). The findings of these studies support a complex genetic basis of SCAD. However, although some of the identified variants have been shown to affect gene expression, most of the genetic associations have remained unexplained.

Environmental factors such as hypertension (HTN) that cause increased vascular pressure and lead to activation of stress- and stretch-induced pathways in aortic smooth muscle cells contribute to increased risk and progression of SCAD. Therefore, factors associated with increased blood pressure (BP) were risk factors for SCAD and AD. GWAS have identified many genetic loci for BP (17), and there were overlapping associated genes between BP, SCAD and AD. Genetic factors may directly influence the structure of the arterial wall or act indirectly through risk factors such as BP, ultimately resulting in an increase in arterial stiffness. The indirect factors involved in the regulatory pathway by which genetic variants influence SCAD and AD risk *via* BP may play roles in SCAD and AD prevention by controlling BP. However, these factors were largely unknown. Identification of indirect factors can help to explain genetic associations.

Despite increasing investment in research and development in the pharmaceutical industry, the rate of success for novel drugs continues to fall. Lower success rates make new therapeutics more expensive, reducing the availability of effective medicines and increasing healthcare costs. Systematically evaluating the genetic evidence in support of potential target-indication pairs is a potential strategy to prioritize development programs. Circulating proteins play key roles in a range of biological processes and represent a major source of druggable targets (18, 19). Recently, published GWAS of circulating proteins have identified a large number of single nucleotide polymorphisms (SNPs) associated with circulating proteins (protein quantitative trait loci, or pQTLs) (20–24). These genetic associations offered the opportunity to systematically test the causal effects of a large number of proteins on diseases. Indeed, the GWAS identified loci containing genes that encode proteins or regulate the expression of proteins involved in arterial wall structure and/or function. Circulating proteins such as TGF β receptor, collagen α1 chain, fibrillin, endotelin-1, ribosome maturation factor, and talin 1 were implicated in the development of SCAD (25). Currently, multiple cytokines, including interleukins, interferon, the tumor necrosis factor superfamily, colony stimulating factor, chemotactic factor, growth factor and so on, have all been demonstrated to play a critical role in SCAD.

Identification of functional variants in these genetic loci for BP, SCAD and AD will increase our understanding of the pathological mechanisms. The recently developed two-sample Mendelian randomization (MR) methods based on summary statistics provide feasible ways to integrate omics data from independent GWAS, including pQTL studies (26–28). This study represents an effort to identify potential proteins or biological pathways implicated in the development of SCAD and AD through BP regulation based on their shared genetic basis.

## Materials and Methods

### Determination of Spontaneous Coronary Artery Dissection and Blood Pressure Associated SNPs

**FIGURE 1:**
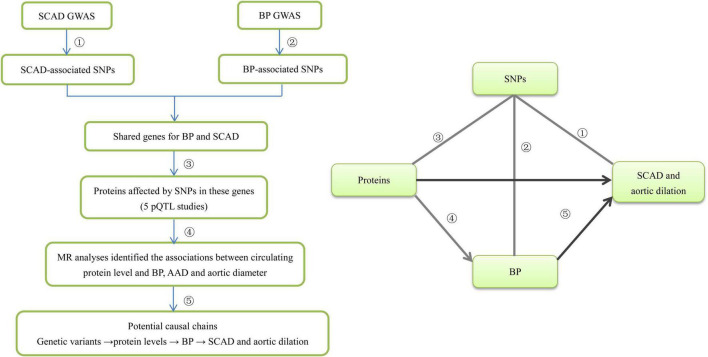
The flowchart of the study design. We designed this study to explore potential risk factors such as circulating proteins for SCAD. This study comprised several steps of analysis. In the first step we identified SCAD-associated genes from five GWASs. Second, we identified BP-associated SNPs in the SCAD-associated genes from BP GWAS and then identified the shared genes for BP and SCAD. Third, we looked for pQTLs inside these genes using data from five pQTL studies. Fourth, for circulating protein levels that were significantly affected by both SCAD-associated and BP-associated SNPs, we applied four MR methods (IVW, MR-Egger, MR-PRESSO, and CAUSE) to examine whether they were genetically associated with BP, AAD, and aortic diameter. Finally, we inferred that genetic variants influence BP by disturbing circulating proteins, and then affecting the risk of SCAD and aortic dilation.

In this study, we determined genetic loci for SCAD from five GWASs (5, 15, 16, 29, 30). Genes and top SNPs in the identified loci were collected from the GWAS papers and related supplementary online files (Figure 1). Specifically, genome-wide summary statistics were obtained from a recent GWAS (15). This GWAS comprised 270 SCAD cases and 5,263 controls of European descent. The raw data used in the present analysis were the downloaded summary results from the initial GWAS, which included association *P*-values of 607,778 genotyped variants with SCAD (15). The SCAD GWAS summary datasets were publicly available at the NHGRI-EBI GWAS Catalog under accession number GCST90000582. SNPs with *P*-values less than 1.0 × 10^–5^ were selected and the SCAD loci were determined. The genome-wide significance level was set to 5.0 × 10^–8^ in this study. The BP loci were identified in a GWAS examined associations between more than 10 million SNPs and systolic BP (SBP) and diastolic BP (DBP) in 317,756 individuals enrolled in the UK Biobank (17). SNPs associated with both BP and SCAD were identified.

### Identification of Circulating Proteins

We carried out pQTL analysis to obtain relevant evidence for the identified SNPs shared by BP and SCAD. The associations between SNPs and circulating protein levels were searched in five pQTL studies. First, the pQTL data of the KORA study which contained associations between 509,946 SNPs and 1,124 proteins were downloaded from the pGWAS Server (21).^1^ The second pQTL study performed genome-wide testing of 10.6 million imputed autosomal variants against levels of 2,994 circulating proteins in 3,301 individuals of European descent from the INTERVAL study (23). The summary data are available at http://www.phpc.cam.ac.uk/ceu/proteins/. The third pQTL study analyzed 83 proteins measured in 3,394 individuals (20).^2^ The fourth pQTL study provided the results of GWAS of 71 high-value cardiovascular disease proteins in 6,861 Framingham Heart Study participants (24). In the fifth pQTL study, 4,137 proteins covering most predicted extracellular proteins were measured in the serum of 5,457 Icelanders over 65 years of age (22). pQTL signals with *P*-values less than 1.0 × 10^–5^ were considered in this study.

### Functional Enrichment Analysis

The purpose of gene ontology (GO) is to unify biological factors while integrating specific definitions, clear structures, and controlled vocabulary into a database (31). GO analysis was performed to explore the biological properties of the genes coding the identified proteins. The Database for Annotation, Visualization and Integrated Discovery (DAVID) database,^3^ an online bioinformatics tool, was used to illustrate the functional enrichment analysis (32). The advantages of DAVID include the detailed analysis and classification of gene and protein functions. A false positive rate less than 0.1 was considered significant.

### MR Analysis on Proteins

To obtain supporting evidence for proteins identified in pQTL analysis, we performed MR analysis to examine the associations between protein levels and BP. The protein data used in these MR analyses were from the pQTL studies described above. The BP GWAS dataset comprised the summary statistics for the association between more than 10 million SNPs and SBP and DBP, which were evaluated in 317,756 individuals enrolled in the UK Biobank (17). This dataset can be downloaded at https://data.broadinstitute.org/alkesgroup/UKBB/. Summary data necessary to MR analysis were unavailable for SCAD, which kept us from examining the associations between protein levels and SCAD.

Instead, we explored the role of the identified proteins in aortic remodeling, which were highly related to BP. GWAS summary data of aortic aneurysm and dissection (AAD) (ICD-10 code: I71), which contains 452,264 individuals from the UK Biobank (30), was obtained. These GWAS included 1,470 AAD patients and 450,794 controls. The genome-wide association analysis of the UK Biobank GWAS comprised 62,394 genotyped variants and 9,113,133 imputed variants that passed quality control. The summary data for AAD can be found by searching “Aortic aneurysm and dissection” at http://geneatlas.roslin.ed.ac.uk/downloads/. In addition, summary data from GWAS of ascending aortic (AA) and descending aortic (DA) diameters were obtained. AA and DA diameters were evaluated in cardiac magnetic resonance images from the UK Biobank by a deep learning model (33).

We employed the weighted median, inverse-variance weighted (IVW) MR (34), MR-Egger (35), MR pleiotropy residual sum and outlier (MR-PRESSO) (36) and the Causal Analysis Using Summary Effect estimates (CAUSE) (37) methods to test for potential causal relationships between circulating protein levels and BP and SCAD. The inverse-variance weighted method combines the ratio estimates from each IV in a meta-analysis model (34). If the associations with circulating protein levels were to lead to horizontal pleiotropy, the intercept from MR-Egger would be expected to differ from zero (35). In such cases, we interpreted the coefficient from MR-Egger as being the more valid causal estimate. Conversely, in the absence of statistical evidence for horizontal pleiotropy from the intercept on MR-Egger, we used the IVW MR analysis as it retains greater power. The IVW MR and MR-Egger analyses were performed by using the MendelianRandomization R package (38). We also detected horizontal pleiotropy and outlier-corrected causal estimation by using MR-PRESSO tests (36). The outlier test in MR-PRESSO is the procedure to test for the MR assumption of no pleiotropy. The source code and documents for MR-PRESSO are available at https://github.com/rondolab/MR-PRESSO. The default parameters were used for the MR-PRESSO analysis.

In the pQTL summary data, SNPs with a *P*-value less than 1.0 × 10^–5^ were selected as potential instrumental variables. The selection criterion was set to 1.0 × 10^–5^ for the two pQTL studies because 5.0 × 10^–8^ would lead to too few instrumental variables. We clumped SNPs (LD *r*^2^ < 0.01 within 10,000 kb) based on data from Europeans from the 1000 Genomes project using the “clump_data” function in the R package TwoSampleMR to select independent instrumental variables. The effect allele of each SNP in the BP GWAS and pQTL studies was manually checked for consistency.

For proteins that passed the three MR tests, we applied the CAUSE method to account for horizontal pleiotropic effects by pathways other than those considered in the multivariable approach. CAUSE is a Mendelian randomization accounting for correlated and uncorrelated horizontal pleiotropic effects using genome-wide summary statistics (37). Data used in the CAUSE analysis were from pQTL studies and GWAS described above. We used 1,000,000 genetic variants to estimate the nuisance parameters. Other parameters were left as their defaults in the CAUSE analysis.

## Results

### The Spontaneous Coronary Artery Dissection-Associated Loci

Based on published GWAS papers, we collected up to 162 SCAD-associated genes (Supplementary Table 1). Genes such as *FBN1* and *PHACTR1* were confirmed in several GWAS. The most recent GWAS published at Nature Communication identified many novel genes and confirmed previously identified genes. In this GWAS, we selected SNPs in 13 SCAD-associated loci (Table 1). The top signals were located in 1q21.2, in which 149 SNPs showed significant associations with SCAD (*P* < 5.0 × 10^–8^). The top SNP associated with SCAD in 1q21.2 was rs12740679 (*P* = 2.19 × 10^–12^). There were fewer genome-wide significant SNPs in other selected loci. Five SNPs in 12q13.3 and one SNP in 6p24.1 were significantly associated with SCAD. For the remaining ten selected loci, no SNPs reached the genome-wide significance level. For 2p23.2, 4q34.3, 5q23.2, and 12q21.33, only one SNP reached 1.0 × 10^–5^ in each locus. In 6q25.3, 11 SNPs passed 1.0 × 10^–5^. The top SNP in 6q25.3 was rs78349783 (1.03 × 10^–6^).

**TABLE 1:** The SCAD loci collected from GWAS.

Study	Loci	Number of SNPs with *P*-value < 1.0E-5	Number of SNPs with *P*-value < 5.0E-8	Top SNP	
				rs ID	*P*-value
(15)	1p32.1	7	0	rs11207415	3.23E-06
	1q21.2	291	149	rs12740679	2.19E-12
	2p23.2	1	0	rs4535004	8.32E-06
	2q33.2	3	0	rs78377252	2.43E-06
	4q34.3	1	0	rs2715408	7.64E-06
	5q23.2	1	0	rs17839701	9.94E-06
	6p24.1	5	1	rs9349379	4.36E-08
	6q22.1	3	0	rs7775726	6.94E-06
	6q25.3	11	0	rs78349783	1.03E-06
	12q13.3	14	5	rs11172113	2.63E-08
	12q21.33	1	0	rs2722224	6.71E-06
	12q23.2	3	0	rs1014675	5.45E-06
	21q22.11	3	0	rs28451064	1.19E-07
(16)	1q21.3	–	1	rs4970935	3.26E-16
	1q24.2	1	–	rs6700122	1.40E-06
	3q22.3	1	–	rs189056	7.53E-06
	4q34.3	1	–	rs79603310	7.32E-05
	6p24.1	–	1	rs9349379	4.59E-14
	8q24.3	1	–	rs10095937	5.74E-05
	12q13.3	–	1	rs11172113	1.42E-13
	15q21.1	–	1	rs2015637	2.12E-09
	16q24.1	1	–	rs67049921	5.40E-05
	21q22.11	–	1	rs28451064	1.09E-09
(39)	1p32.1	1	–	rs12402265	2.30E-07
	2q32.1	1	–	rs6741522	2.29E-06
	4q12	–	1	rs6820391	2.36E-08
	6p24.1	–	1	rs9349379	1.00E-11
	12q13.3	2	–	rs11172113	1.90E-07
(5)	3p22.1	–	1	rs2272007	1.15E-09
	12q13.3	–	1	rs11172113	2.74E-08
	15q21.1	–	1	rs1042078	1.08E-10
(4)	15q21.1	–	5	rs2118181	5.90E-12
	Total	353	170	–	–

### Spontaneous Coronary Artery Dissection-Associated Genes Shared by Blood Pressure

We selected 230 thousand SNPs inside the 162 SCAD-associated genes according to the UCSC database. Then we looked up associations between these SNPs and BP in GWAS summary data. We found 204 and 228 SNPs that were significantly associated with SBP and DBP (*P* < 5.0 × 10^–8^), respectively. These SNPs were located in 11 genes, including *JAG1*, *ERI1*, *ULK4*, *THSD4*, *CMIP*, *COL4A2*, *FBN1*, *FAM76B*, *FGGY, NUS1*, and *HNF4G*. A total of 111 and 114 SNPs in the *ULK4* gene were associated with SBP and DBP (*P* < 5.0 × 10^–8^), respectively. SNPs in *JAG1* and *ERI1* both associated with SBP and DBP. SNPs in *FGGY*, *HNF4G* and *NUS1* were associated with SBP; SNPs in *CMIP*, *COL4A2*, *FAM76B*, *FBN1*, and *THSD4* were associated with DBP. These SNPs were located in SCAD-associated genes, and therefore were considered to be shared SNPs for SCAD and BP. We next searched whether these SNPs were associated with circulating proteins.

### Potential Proteins Related to Blood Pressure and Spontaneous Coronary Artery Dissection

We looked for pQTL signals in five studies. We found significant signals in three studies (20, 21, 23) for the shared SNPs (Table 2). A total of 49 pQTL signals were identified. The SNPs were located in *FAM76B*, *COL4A2*, *JAG1*, *ITSN1*, *ERI1*, and *CMIP*. The regulated proteins were encoded by 13 genes, including *MMP10*, *IL6R*, *FIGF*, *MMP1*, *CTSB*, *IGHG1*, *DSG2*, *TTC17*, *RETN*, *POMC*, *SCARF2*, *RELT*, and *GALNT16*. BP-associated SNPs in *ERI1* were strongly associated with circulating cathepsin B (*CTSB*), and evidence was found in KORA and INTERVAL studies (21, 23).

**TABLE 2:** Proteins related to BP-associated SNPs.

SNP	Gene	CHR	Position (hg19)	GWAS		pQTL		
				*P*-value	Trait	*P*-value	Protein	Study
rs11776838	*ERI1*	8	8794801	1.22E-08	SBP	5.23E-05	*CTSB*	(21)
rs11776838	*ERI1*	8	8794801	1.33E-12	DBP	5.23E-05	*CTSB*	(21)
rs2186739	*FAM76B*	11	95327374	8.55E-11	DBP	2.48E-05	*IGHG1*	(21)
rs2186739	*FAM76B*	11	95327374	8.55E-11	DBP	7.98E-05	*DSG2*	(21)
rs2156468	*FAM76B*	11	95327412	4.18E-08	DBP	2.41E-05	*IGHG1*	(21)
rs2508895	*FAM76B*	11	95321747	5.04E-11	DBP	2.34E-04	*MMP10*	(20)
rs2254626	*FAM76B*	11	95327169	8.78E-11	DBP	8.21E-04	*MMP10*	(20)
rs2186739	*FAM76B*	11	95327374	8.55E-11	DBP	8.45E-04	*MMP10*	(20)
rs12323265	*COL4A2*	13	111014716	1.50E-08	DBP	4.55E-04	*IL6R*	(20)
rs6077929	*JAG1*	20	10819210	6.67E-12	DBP	6.27E-04	*FIGF*	(20)
rs2251854	*ITSN1*	21	35056247	3.99E-06	DBP	8.58E-04	*MMP1*	(20)
rs7281625	*ITSN1*	21	35066836	4.89E-06	DBP	9.74E-04	*MMP1*	(20)
rs475259	*ERI1*	8	8610267	2.68E-09	SBP	4.68E-05	*TTC17*	(23)
rs475259	*ERI1*	8	8610267	6.46E-10	DBP	4.68E-05	*TTC17*	(23)
rs28446104	*ERI1*	8	8795901	1.10E-11	DBP	2.95E-05	*RETN*	(23)
rs28446104	*ERI1*	8	8795901	1.10E-11	DBP	2.75E-05	*CTSB*	(23)
rs7819827	*ERI1*	8	8797055	4.74E-08	SBP	4.90E-05	*CTSB*	(23)
rs7819827	*ERI1*	8	8797055	3.34E-12	DBP	4.90E-05	*CTSB*	(23)
rs7844374	*ERI1*	8	8798684	4.25E-08	SBP	3.80E-05	*CTSB*	(23)
rs7844374	*ERI1*	8	8798684	2.26E-12	DBP	3.80E-05	*CTSB*	(23)
rs6601274	*ERI1*	8	8799059	2.03E-08	SBP	2.88E-05	*RETN*	(23)
rs6601274	*ERI1*	8	8799059	2.03E-08	SBP	3.89E-05	*CTSB*	(23)
rs6601274	*ERI1*	8	8799059	3.31E-12	DBP	3.89E-05	*CTSB*	(23)
rs6601274	*ERI1*	8	8799059	3.31E-12	DBP	2.88E-05	*RETN*	(23)
rs7837026	*ERI1*	8	8801692	5.80E-09	SBP	3.80E-05	*CTSB*	(23)
rs7837026	*ERI1*	8	8801692	5.80E-09	SBP	3.55E-05	*RETN*	(23)
rs7837026	*ERI1*	8	8801692	3.79E-13	DBP	3.55E-05	*RETN*	(23)
rs7837026	*ERI1*	8	8801692	3.79E-13	DBP	3.80E-05	*CTSB*	(23)
rs7823898	*ERI1*	8	8803487	5.25E-11	DBP	7.59E-06	*RETN*	(23)
rs12930850	*CMIP*	16	81602212	7.24E-09	DBP	3.24E-06	*POMC*	(23)
rs12929303	*CMIP*	16	81602264	3.47E-09	DBP	3.24E-06	*POMC*	(23)
rs11649004	*CMIP*	16	81602499	9.12E-09	DBP	1.66E-06	*POMC*	(23)
rs11644375	*CMIP*	16	81602681	8.88E-09	DBP	5.50E-06	*POMC*	(23)
rs8045875	*CMIP*	16	81603235	6.24E-09	DBP	5.25E-06	*POMC*	(23)
rs8045100	*CMIP*	16	81603400	5.80E-09	DBP	3.09E-06	*POMC*	(23)
rs11640346	*CMIP*	16	81603848	6.83E-09	DBP	6.03E-06	*POMC*	(23)
rs12931242	*CMIP*	16	81604778	6.11E-09	DBP	2.09E-06	*POMC*	(23)
rs7198940	*CMIP*	16	81605559	2.40E-08	DBP	4.27E-06	*POMC*	(23)
rs12918645	*CMIP*	16	81606569	2.55E-08	DBP	4.07E-06	*POMC*	(23)
rs12924701	*CMIP*	16	81607756	4.18E-09	DBP	3.31E-06	*POMC*	(23)
rs4483835	*CMIP*	16	81607833	4.81E-09	DBP	2.51E-06	*POMC*	(23)
rs7196548	*CMIP*	16	81609774	6.20E-09	DBP	1.91E-06	*POMC*	(23)
rs7199293	*CMIP*	16	81614892	4.29E-08	DBP	1.78E-05	*POMC*	(23)
rs6134025	*JAG1*	20	10743782	4.64E-10	DBP	2.29E-05	*SCARF2*	(23)
rs75612301	*JAG1*	20	10744573	5.36E-10	DBP	4.07E-05	*RELT*	(23)
rs75612301	*JAG1*	20	10744573	5.36E-10	DBP	2.00E-05	*SCARF2*	(23)
rs6134028	*JAG1*	20	10744714	1.76E-09	DBP	3.09E-05	*SCARF2*	(23)
rs1009757	*JAG1*	20	10828640	2.67E-14	DBP	3.72E-05	*GALNT16*	(23)
rs6074192	*JAG1*	20	10829248	3.28E-14	DBP	4.17E-05	*GALNT16*	(23)

*CHR, Chromosome; GWAS, Genome-wide association study; QTL, Quantitative trait locus; SNP, Single nucleotide polymorphism.*

### Functional Enrichment Analysis

To examine the potential biological functions of the proteins affected by SCAD and BP-associated SNPs, we performed GO analysis in the DAVID database. Among the 11 shared genes, *COL4A2*, *THSD4*, and *FBN1* were related to extracellular matrix components (*P* = 1.10 × 10^–3^) and proteinaceous extracellular matrix (*P* = 8.60 × 10^–7^). The 13 BP- and SCAD-associated proteins were enriched in specific GO biological process, cellular component and molecular function terms (false discovery rate < 0.1), including cellular components such as extracellular space (*P* = 2.70 × 10^–3^); biological process such as collagen metabolic process (*P* = 5.70 × 10^–7^), multicellular organism metabolic process (*P* = 6.90 × 10^–7^); molecular function such as serine-type peptidase activity (*P* = 6.50 × 10^–4^), serine hydrolase activity (*P* = 6.70 × 10^–4^) and endopeptidase activity (*P* = 3.30 × 10^–3^) (Table 3). Proteins encoded by *IGHG1*, *MMP1*, *MMP10*, *CTSB*, *POMC*, *RETN*, and *IL6R* may have functional roles in BP regulation and the development of SCAD.

**TABLE 3:** Potential biological functions of proteins regulated by BP and SCAD-associated SNPs.

Catalog	Term	Gene	*P*-value	Benjamini
Molecular function	Serine-type endopeptidase activity	*IGHG1, MMP1, MMP10, CTSB*	4.90E-04	1.50E-02
Molecular function	Serine-type peptidase activity	*IGHG1, MMP1, MMP10, CTSB*	6.50E-04	1.50E-02
Molecular function	Serine hydrolase activity	*IGHG1, MMP1, MMP10, CTSB*	6.70E-04	1.50E-02
Molecular function	Endopeptidase activity	*IGHG1, MMP1, MMP10, CTSB*	3.30E-03	5.60E-02
Biological process	Collagen metabolic process	*MMP1, RETN, IL6R, MMP10, CTSB*	5.70E-07	1.80E-04
Biological process	Multicellular organismal macromolecule metabolic process	*MMP1, RETN, IL6R, MMP10, CTSB*	6.90E-07	1.80E-04
Biological process	Multicellular organism metabolic process	*MMP1, RETN, IL6R, MMP10, CTSB*	1.20E-06	2.20E-04
Cellular component	Extracellular space	*POMC, IGHG1, RETN, IL6R, MMP10, CTSB*	2.70E-03	6.20E-02

### Proteins Causally Associated With Blood Pressure and Aortic Remodeling

**FIGURE 2:**
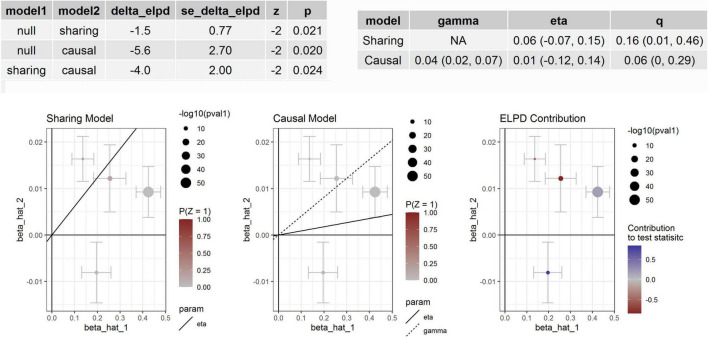
Association between circulating cathepsin B and SBP. Effect-size estimates and variant-level contribution to CAUSE test statistics for circulating cathepsin B and SBP.

We tested whether the seven proteins were genetically associated with BP using several MR methods (Table 4). We found that the association between circulating cathepsin B level and DBP was significant in MR-Egger analyses using data from KORA and INTERVAL studies. Significant association between circulating cathepsin B level and DBP was also found in the weighted median analysis using data from INTERVAL study. After adjusting for outliers in MR-PRESSO analysis, the associations between circulating cathepsin B level and DBP was marginal significant (*P* = 0.0580). Significant association between circulating cathepsin B level and SBP was found in the weighted median analysis using data from INTERVAL study. Furthermore, we corrected for correlated and uncorrelated horizontal pleiotropy using the CAUSE method and retained an indication for the causal effect of cathepsin B on BP (Figure 2). By using INTERVAL data, we found that the causal model was significantly better than the null and sharing models for SBP (*P* = 0.020 and 0.024, respectively). The instrumental variables used in the MR analyses were presented in Supplementary Table 2.

**TABLE 4:** The causation between circulating cathepsin B level and BP and aortic diameter.

Trait	pQTL study	Weighted median			IVW			MR-Egger			MR-PRESSO		
		Beta	SE	*P*-value	Beta	SE	*P*-value	Beta	SE	*P*-value	Beta	SE	*P*-value
DBP	KORA	–0.0901	0.0405	0.0263	–0.0829	0.0720	0.2496	–0.3668	0.0822	8.15 × 10^–6^	–0.0883	0.0757	0.3636
	INTERVAL	–0.1499	0.0453	9.25 × 10^–4^	–0.0688	0.0520	0.1864	–0.3057	0.0630	1.23 × 10^–6^	–0.0937	0.0451	0.0580
SBP	KORA	–0.0131	0.0057	0.0213	–0.0053	0.0098	0.5909	–0.0437	0.0196	0.0257	–0.0053	0.0098	0.6194
	INTERVAL	–0.0214	0.0064	8.56 × 10^–4^	–0.0120	0.0077	0.1186	–0.0197	0.0186	0.2908	–0.0120	0.0077	0.1381
AA	KORA	–0.0247	0.0135	0.0676	–0.0239	0.0148	0.1058	–0.0950	0.0280	6.81 × 10^–4^	–0.0238	0.0147	0.1811
	INTERVAL	–0.0596	0.0152	8.34 × 10^–5^	–0.0397	0.0136	3.63 × 10^–3^	–0.0234	0.0324	0.4712	–0.0397	0.0136	9.38 × 10^–3^
DA	KORA	–0.0232	0.0105	0.0282	–0.0221	0.0091	0.0153	–0.0340	0.0248	0.1706	–0.0221	0.0040	5.18 × 10^–3^
	INTERVAL	–0.0249	0.0134	0.0629	–0.0106	0.0134	0.4293	–0.0521	0.0303	0.0852	–0.0106	0.0134	0.4397

*BP, Blood pressure; IVW, inverse-variance weighted; MR, Mendelian randomization; MR-PRESSO, MR pleiotropy residual sum and outlier; SE, Standard error.*

**FIGURE 3:**
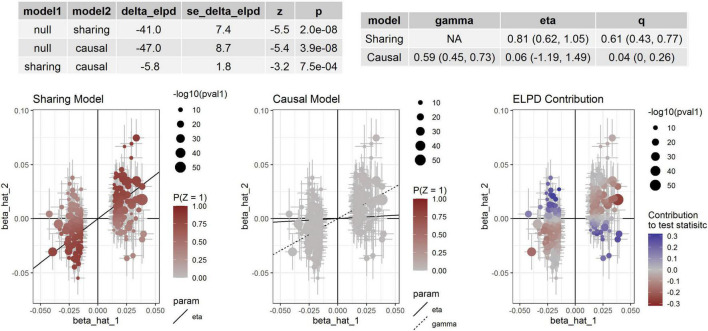
Association between DBP and AA diameter. Effect-size estimates and variant-level contribution to CAUSE test statistics for DBP and AA diameter.

For aortic remodeling, we first examined the association between BP and AAD and aortic diameters by MR analysis (Table 5). Significant associations between DBP and AAD, AA and DA diameters were found. For SBP, significant association between SBP and AA diameter were found in weighted median analysis and MR-PRESSO analysis. Marginal significant association between DBP and AAD was found in the CAUSE analysis (*P* = 0.049). Significant association between DBP and AA diameter was found in the CAUSE analysis (*P* = 7.48 × 10^–4^) (Figure 3). The instrumental variables used in the MR analyses were presented in Supplementary Table 3.

**TABLE 5:** The causation between BP and AAD and aortic diameter.

X	Y	Method	Beta	SE	*P*-value
DBP	AAD	Weighted median	0.0028	0.0008	3.83E-04
DBP	AAD	IVW	0.0025	0.0006	1.26E-05
DBP	AAD	MR-Egger	0.0019	0.0020	0.3538
DBP	AAD	MR-PRESSO	0.0023	0.0005	4.99E-05
DBP	AA diameter	Weighted median	0.4463	0.0511	2.41E-18
DBP	AA diameter	IVW	0.4841	0.0560	5.73E-18
DBP	AA diameter	MR-Egger	0.5877	0.1965	2.78E-03
DBP	AA diameter	MR-PRESSO	0.4477	0.0446	8.81E-19
DBP	DA diameter	Weighted median	0.1732	0.0351	8.08E-07
DBP	DA diameter	IVW	0.1413	0.0355	7.03E-05
DBP	DA diameter	MR-Egger	0.0829	0.1143	0.4681
DBP	DA diameter	MR-PRESSO	0.1599	0.0299	1.78E-07
SBP	AAD	Weighted median	0.0010	0.0008	0.1987
SBP	AAD	IVW	0.0005	0.0006	0.4545
SBP	AAD	MR-Egger	0.0028	0.0021	0.1851
SBP	AAD	MR-PRESSO	0.0006	0.0006	0.3399
SBP	AA diameter	Weighted median	0.1634	0.0554	3.16E-03
SBP	AA diameter	IVW	0.0729	0.0734	0.3204
SBP	AA diameter	MR-Egger	0.0480	0.2403	0.8416
SBP	AA diameter	MR-PRESSO	0.1969	0.0498	1.19E-04
SBP	DA diameter	Weighted median	0.0454	0.0473	0.3374
SBP	DA diameter	IVW	–0.0425	0.0574	0.4592
SBP	DA diameter	MR-Egger	–0.1035	0.1878	0.5815
SBP	DA diameter	MR-PRESSO	0.0230	0.0440	0.6018

We then examined the association between circulating cathepsin B level and AAD and aortic diameters by MR analysis (Table 4). Significant association between circulating cathepsin B level and AAD was not found. We found that significant associations between circulating cathepsin B level and AA diameter in weighted median (*P* = 8.34 × 10^–5^), IVW (*P* = 3.63 × 10^–3^) and MR-PRESSO (*P* = 9.38 × 10^–3^) analyses by using the INTERVAL data. We found that nominal associations between circulating cathepsin B level and AA (*P* = 0.038) and DA (*P* = 0.067) diameters in the CAUSE analysis by using the INTERVAL data. The instrumental variables used in the MR analyses were presented in Supplementary Table 4.

## Discussion

This study searched for potential SCAD-related proteins that may act through BP regulation by integrative analysis of data from AAD, aortic diameters and BP GWAS and pQTL studies. We identified shared genetic variants and circulating proteins for BP and SCAD. Additionally, causal associations between BP and AAD and aortic diameters were detected. Moreover, we found proteins associated with BP and aortic diameters and highlighted circulating cathepsin B that was causally associated with BP and aortic diameters.

Genetic factors play roles in the etiology of SCAD. The findings of genetic association studies provided new biological leads for further mechanistic investigation of arterial pathobiology. In this study, we collected SCAD susceptibility genes in GWAS-identified loci and identified BP-associated SNPs in *JAG1*, *ERI1*, *ULK4*, *THSD4*, *CMIP*, *COL4A2*, *FBN1*, *FAM76B*, *FGGY, NUS1*, and *HNF4G*. The shared genetic variants in these genes both disturbed BP regulation and affected SCAD risk. As HTN is a causal risk factor for SCAD, the shared genetic variants may first regulate BP and then affect SCAD risk. The shared genes were related to extracellular matrix components, indicating that the effect of the genetic variants on BP regulation and their effects on SCAD risk may act through extracellular matrix components. The regulatory effects of genetic variants on BP should also be indirect. Therefore, identification of the indirect factors in the regulatory pathway was the key to elucidating the genetic association. Circulating proteins are critical factors implicated in the development of SCAD (25). We found that the shared genetic variants were strongly associated with many circulating proteins and that the associated proteins have specific biological functions. We found *IGHG1*, *MMP1*, *MMP10*, *CTSB*, *POMC*, *RETN*, and *IL6R*, which were related to endopeptidase activity, collagen metabolic process and extracellular space. Among these associated protein-coding genes, *MMP1*, *CTSB*, *POMC*, *RETN*, and *IL6R* are known to be associated with SCAD (40, 41) or other cardiovascular diseases (42–44). As circulating proteins are druggable targets, identification of the regulated proteins in the pathway was helpful for understanding the relationship between BP and SCAD and valuable for SCAD prevention.

*CTSB* encodes cathepsin B, which is related to the activated TLR4 signaling and degradation of the extracellular matrix pathway and participates in intracellular degradation and turnover of proteins (45). Cathepsin B could be involved in the intracellular processing of prorenin that is locally synthesized or taken up from the extracellular compartment (46). The circulating level of cathepsin B was associated with cardiovascular events and mortality (42). However, the association between circulating levels of cathepsin B and BP or HTN has not been reported. Renin is the key enzyme of the renin-angiotensin system, which is involved in BP regulation. Cathepsin B has been proposed as a prorenin processing enzyme because of its colocalization with renin and its ability to activate prorenin (47–49). In this study, we found that BP-associated SNPs were strongly associated with cathepsin B levels in two independent studies. The association between cathepsin B level and BP, AAD and aortic diameters was identified by several MR analysis methods and was validated by independent data. Therefore, cathepsin B was associated with BP and may be involved in aortic remodeling.

The present study has some potential limitations. First, although we found significant associations between circulating protein levels and BP and aortic diameters, the direct relationships between circulating protein levels and SCAD were not detected. This was because that necessary summary data (e.g., beta and se) of the SCAD GWAS were unavailable and the sample size of the AAD GWAS was small (*n* = 1,470), in which the necessary summary data were available. Second, most of the associations between the identified SNPs and proteins have not been validated in another independent sample because of lack of data. Finally, the functional relationships have not been validated by technical and biological experiments. Further *in vitro* studies are needed to determine their functions in related cell lines such as vascular smooth muscle cells.

## Conclusion

In summary, the present study identified genetic variants shared by BP and SCAD and found that the associated SNPs may have strong effects on circulating protein levels. Therefore, this study found circulating proteins that were pleiotropically associated with BP and SCAD. Circulating cathepsin B was associated with BP and aortic diameters and therefore may play important roles in BP regulation and aortic remodeling. The findings suggested that genetic variants of BP genes may have regulatory potential to affect circulating proteins involved in BP regulation and ultimately affect risk of cardiovascular disease.

## Data Availability Statement

The datasets presented in this study can be found in online repositories. The names of the repository/repositories and accession number(s) can be found in the article/Supplementary Material.

## Author Contributions

TC and LC designed this study. TC, XY, and ZQ collected the data. TC was responsible for the statistical analysis. MT and TC wrote the draft. MT, XL, and LC revised this draft. LC finalized this manuscript. All authors read and approved the final manuscript.

## Conflict of Interest

The authors declare that the research was conducted in the absence of any commercial or financial relationships that could be construed as a potential conflict of interest.

## Publisher’s Note

All claims expressed in this article are solely those of the authors and do not necessarily represent those of their affiliated organizations, or those of the publisher, the editors and the reviewers. Any product that may be evaluated in this article, or claim that may be made by its manufacturer, is not guaranteed or endorsed by the publisher.

## Footnotes

1
http://metabolomics.helmholtz-muenchen.de/pgwas/index.php?task=download

2
https://zenodo.org/record/264128#.X3u5ga95uUk

3
https://david.ncifcrf.gov/

## References

[1] Sampson UK Norman PE Fowkes FG Aboyans V Song Y Harrell FE Jr. Estimation of global and regional incidence and prevalence of abdominal aortic aneurysms 1990 to 2010. *Glob Heart.* (2014) 9:159–70. 10.1016/j.gheart.2013.12.009 25432125

[2] Sampson UK Norman PE Fowkes FG Aboyans V Yanna S Harrell FE Jr. Global and regional burden of aortic dissection and aneurysms: mortality trends in 21 world regions, 1990 to 2010. *Glob Heart.* (2014) 9:171–180e10. 10.1016/j.gheart.2013.12.010 25432126

[3] Lee B Godfrey M Vitale E Hori H Mattei MG Sarfarazi M Linkage of marfan syndrome and a phenotypically related disorder to two different fibrillin genes. *Nature.* (1991) 352:330–4. 10.1038/352330a0 1852206

[4] LeMaire SA McDonald ML Guo DC Russell L Miller CC III Johnson RJ Genome-wide association study identifies a susceptibility locus for thoracic aortic aneurysms and aortic dissections spanning FBN1 at 15q21.1. *Nat Genet.* (2011) 43:996–1000. 10.1038/ng.934 21909107 PMC3244938

[5] Guo DC Grove ML Prakash SK Eriksson P Hostetler EM LeMaire SA Genetic variants in LRP1 and ULK4 are associated with acute aortic dissections. *Am J Hum Genet.* (2016) 99:762–9. 10.1016/j.ajhg.2016.06.034 27569546 PMC5011062

[6] van ’t Hof FN Ruigrok YM Lee CH Ripke S Anderson G de Andrade M Shared genetic risk factors of intracranial, abdominal, and thoracic aneurysms. *J Am Heart Assoc.* (2016) 5:e002603. 10.1161/JAHA.115.002603 27418160 PMC5015357

[7] Nienaber CA Clough RE Sakalihasan N Suzuki T Gibbs R Mussa F Aortic dissection. *Nat Rev Dis Primers.* (2016) 2:16053. 10.1038/nrdp.2016.53 27440162

[8] Kaadan MI MacDonald C Ponzini F Duran J Newell K Pitler L Prospective cardiovascular genetics evaluation in spontaneous coronary artery dissection. *Circ Genom Precis Med.* (2018) 11:e001933. 10.1161/CIRCGENETICS.117.001933 29650765

[9] Solomonica A Bagur R Choudhury T Lavi S. Familial spontaneous coronary artery dissection and the SMAD-3 mutation. *Am J Cardiol.* (2019) 124:313–5. 10.1016/j.amjcard.2019.04.035 31085000

[10] Verstraeten A Perik M Baranowska AA Meester JAN Van Den Heuvel L Bastianen J Enrichment of rare variants in Loeys-Dietz syndrome genes in spontaneous coronary artery dissection but not in severe fibromuscular dysplasia. *Circulation.* (2020) 142:1021–4. 10.1161/CIRCULATIONAHA.120.045946 32897753

[11] Adlam D Olson TM Combaret N Kovacic JC Iismaa SE Al-Hussaini A Association of the PHACTR1/EDN1 genetic locus with spontaneous coronary artery dissection. *J Am Coll Cardiol.* (2019) 73:58–66. 10.1016/j.jacc.2018.09.085 30621952 PMC10403154

[12] Grond-Ginsbach C Böckler D Newton-Cheh C. Pathogenic TSR1 gene variants in patients with spontaneous coronary artery dissection. *J Am Coll Cardiol.* (2019) 74:177–8. 10.1016/j.jacc.2019.06.005 31296288

[13] Sun Y Chen Y Li Y Li Z Li C Yu T Association of TSR1 variants and spontaneous coronary artery dissection. *J Am Coll Cardiol.* (2019) 74:167–76. 10.1016/j.jacc.2019.04.062 31296287

[14] Turley TN Theis JL Sundsbak RS Evans JM O’Byrne MM Gulati R Rare missense variants in TLN1 Are associated with familial and sporadic spontaneous coronary artery dissection. *Circ Genom Precis Med.* (2019) 12:e002437. 10.1161/CIRCGEN.118.002437 30888838 PMC6625931

[15] Saw J Yang ML Trinder M Tcheandjieu C Xu C Starovoytov A Chromosome 1q21.2 and additional loci influence risk of spontaneous coronary artery dissection and myocardial infarction. *Nat Commun.* (2020) 11:4432. 10.1038/s41467-020-17558-x 32887874 PMC7474092

[16] Turley TN O’Byrne MM Kosel ML de Andrade M Gulati R Hayes SN Identification of susceptibility loci for spontaneous coronary artery dissection. *JAMA Cardiol.* (2020) 5:1–10. 10.1001/jamacardio.2020.0872 32374345 PMC7203673

[17] Evangelou E Warren HR Mosen-Ansorena D Mifsud B Pazoki R Gao H Genetic analysis of over 1 million people identifies 535 new loci associated with blood pressure traits. *Nat Genet.* (2018) 50:1412–25. 10.1038/s41588-018-0205-x 30224653 PMC6284793

[18] Imming P Sinning C Meyer A. Drugs, their targets and the nature and number of drug targets. *Nat Rev Drug Discov.* (2006) 5:821–34. 10.1038/nrd2132 17016423

[19] Santos R Ursu O Gaulton A Bento AP Donadi RS Bologa CG A comprehensive map of molecular drug targets. *Nat Rev Drug Discov.* (2017) 16:19–34. 10.1038/nrd.2016.230 27910877 PMC6314433

[20] Folkersen L Fauman E Sabater-Lleal M Strawbridge RJ Frånberg M Sennblad B Mapping of 79 loci for 83 plasma protein biomarkers in cardiovascular disease. *PLoS Genet.* (2017) 13:e1006706. 10.1371/journal.pgen.1006706 28369058 PMC5393901

[21] Suhre K Arnold M Bhagwat AM Cotton RJ Engelke R Raffler J Connecting genetic risk to disease end points through the human blood plasma proteome. *Nat Commun.* (2017) 8:14357. 10.1038/ncomms14357 28240269 PMC5333359

[22] Emilsson V Ilkov M Lamb JR Finkel N Gudmundsson EF Pitts R Co-regulatory networks of human serum proteins link genetics to disease. *Science.* (2018) 361:769–73. 10.1126/science.aaq1327 30072576 PMC6190714

[23] Sun BB Maranville JC Peters JE Stacey D Staley JR Blackshaw J Genomic atlas of the human plasma proteome. *Nature.* (2018) 558:73–9. 10.1038/s41586-018-0175-2 29875488 PMC6697541

[24] Yao C Chen G Song C Keefe J Mendelson M Huan T Genome-wide mapping of plasma protein QTLs identifies putatively causal genes and pathways for cardiovascular disease. *Nat Commun.* (2018) 9:3268. 10.1038/s41467-018-05512-x 30111768 PMC6093935

[25] Di Fusco SA Rossini R Zilio F Pollarolo L di Uccio FS Iorio A Spontaneous coronary artery dissection: overview of pathophysiology. *Trends Cardiovasc Med.* (2021) 32:92–100. *, 10.1016/j.tcm.2021.01.002 33453416

[26] Gamazon ER Wheeler HE Shah KP Mozaffari SV Aquino-Michaels K Carroll RJ A gene-based association method for mapping traits using reference transcriptome data. *Nat Genet.* (2015) 47:1091–8. 10.1038/ng.3367 26258848 PMC4552594

[27] Gusev A Ko A Shi H Bhatia G Chung W Penninx BW Integrative approaches for large-scale transcriptome-wide association studies. *Nat Genet.* (2016) 48:245–52. 10.1038/ng.3506 26854917 PMC4767558

[28] Zhu Z Zhang F Hu H Bakshi A Robinson MR Powell JE Integration of summary data from GWAS and eQTL studies predicts complex trait gene targets. *Nat Genet.* (2016) 48:481–7. 10.1038/ng.3538 27019110

[29] Bycroft C Freeman C Petkova D Band G Elliott LT Sharp K The UK Biobank resource with deep phenotyping and genomic data. *Nature.* (2018) 562:203–9. 10.1038/s41586-018-0579-z 30305743 PMC6786975

[30] Canela-Xandri O Rawlik K Tenesa A. An atlas of genetic associations in UK biobank. *Nat Genet.* (2018) 50:1593–9. 10.1038/s41588-018-0248-z 30349118 PMC6707814

[31] Ashburner M Ball CA Blake JA Botstein D Butler H Cherry JM Gene ontology: tool for the unification of biology. the gene ontology consortium. *Nat Genet.* (2000) 25:25–9. 10.1038/75556 10802651 PMC3037419

[32] Huang da W Sherman BT Lempicki RA. Systematic and integrative analysis of large gene lists using DAVID bioinformatics resources. *Nat Protoc.* (2009) 4:44–57. 10.1038/nprot.2008.211 19131956

[33] Pirruccello JP Chaffin MD Chou EL Fleming SJ Lin H Nekoui M Deep learning enables genetic analysis of the human thoracic aorta. *Nat Genet.* (2022) 54:40–51. 10.1038/s41588-021-00962-4 34837083 PMC8758523

[34] Burgess S Butterworth A Thompson SG. Mendelian randomization analysis with multiple genetic variants using summarized data. *Genet Epidemiol.* (2013) 37:658–65. 10.1002/gepi.21758 24114802 PMC4377079

[35] Bowden J Davey Smith G Burgess S. Mendelian randomization with invalid instruments: effect estimation and bias detection through egger regression. *Int J Epidemiol.* (2015) 44:512–25. 10.1093/ije/dyv080 26050253 PMC4469799

[36] Verbanck M Chen CY Neale B Do R. Detection of widespread horizontal pleiotropy in causal relationships inferred from mendelian randomization between complex traits and diseases. *Nat Genet.* (2018) 50:693–8. 10.1038/s41588-018-0099-7 29686387 PMC6083837

[37] Morrison J Knoblauch N Marcus JH Stephens M He X. Mendelian randomization accounting for correlated and uncorrelated pleiotropic effects using genome-wide summary statistics. *Nat Genet.* (2020) 52:740–7. 10.1038/s41588-020-0631-4 32451458 PMC7343608

[38] Yavorska OO Burgess S. mendelianrandomization: an R package for performing mendelian randomization analyses using summarized data. *Int J Epidemiol.* (2017) 46:1734–9. 10.1093/ije/dyx034 28398548 PMC5510723

[39] Debette S Kamatani Y Metso TM Kloss M Chauhan G Engelter ST Common variation in PHACTR1 is associated with susceptibility to cervical artery dissection. *Nat Genet.* (2015) 47:78–83. 10.1038/ng.3154 25420145 PMC5824623

[40] Landenhed M Engström G Gottsäter A Caulfield MP Hedblad B Newton-Cheh C Risk profiles for aortic dissection and ruptured or surgically treated aneurysms: a prospective cohort study. *J Am Heart Assoc.* (2015) 4:e001513. 10.1161/JAHA.114.001513 25609416 PMC4330075

[41] Zhuang J Luan P Li H Wang K Zhang P Xu Y The Yin-Yang dynamics of DNA methylation Is the key regulator for smooth muscle cell phenotype switch and vascular remodeling. *Arterioscler Thromb Vasc Biol.* (2017) 37:84–97. 10.1161/ATVBAHA.116.307923 27879253

[42] Ryer EJ Ronning KE Erdman R Schworer CM Elmore JR Peeler TC The potential role of DNA methylation in abdominal aortic aneurysms. *Int J Mol Sci.* (2015) 16:11259–75. 10.3390/ijms160511259 25993294 PMC4463699

[43] Mo XB Lei SF Zhang YH Zhang H. Examination of the associations between m(6)A-associated single-nucleotide polymorphisms and blood pressure. *Hypertens Res.* (2019) 42:1582–9. 10.1038/s41440-019-0277-8 31175347

[44] Zhou X Chen Z Zhou J Liu Y Fan R Sun T. Transcriptome and N6-methyladenosine RNA methylome analyses in aortic dissection and normal human aorta. *Front Cardiovasc Med.* (2021) 8:627380. 10.3389/fcvm.2021.627380 34124185 PMC8193080

[45] Kmietczyk V Riechert E Kalinski L Boileau E Malovrh E Malone B m(6)A-mRNA methylation regulates cardiac gene expression and cellular growth. *Life Sci Alliance.* (2019) 2:e201800233. 10.26508/lsa.201800233 30967445 PMC6458851

[46] Fu Y Dominissini D Rechavi G He C. Gene expression regulation mediated through reversible m*6*A RNA methylation. *Nat Rev Genet.* (2014) 15:293–306. 10.1038/nrg3724 24662220

[47] Griendling KK Minieri CA Ollerenshaw JD Alexander RW. Angiotensin II stimulates NADH and NADPH oxidase activity in cultured vascular smooth muscle cells. *Circ Res.* (1994) 74:1141–8. 10.1161/01.res.74.6.1141 8187280

[48] Dorn LE Lasman L Chen J Xu X Hund TJ Medvedovic M The N(6)-methyladenosine mRNA methylase METTL3 controls cardiac homeostasis and hypertrophy. *Circulation.* (2019) 139:533–45. 10.1161/CIRCULATIONAHA.118.036146 30586742 PMC6340720

[49] Mathiyalagan P Adamiak M Mayourian J Sassi Y Liang Y Agarwal N FTO-dependent N(6)-methyladenosine regulates cardiac function during remodeling and repair. *Circulation.* (2019) 139:518–32. 10.1161/CIRCULATIONAHA.118.033794 29997116 PMC6400591

